# Operationen am Ohr: Die Operationen bei Mittelohreiterungen und ihren Intrakraniellen Complicationen

**Published:** 1904-09

**Authors:** 


**Operationen am Ohr : Die Operationen bei Mittelohreiterungen und ihren Intrakraniellen Complicationen.** Von Dr. B. Heine. Pp. viii., 178. Berlin: S. Karger. 1904.

The methods of operating for the relief of suppurative diseases of the ear and their complications discussed in these pages are those ordinarily carried out in Prof. Lucae's clinic in Berlin. The author, Dr. Heine, is the first assistant in that clinic, which is one of the largest and most famous in existence. No one practising aural surgery can therefore fail to be highly interested in the concise account given, not only of the technique of the various operations as performed there, but also of the conditions which are regarded as the proper indications for each operation and of the details of the after treatment, and in the critical estimate of the value of the operations, which is in each case appended.

Nearly two-thirds of the book are devoted to the consideration in detail of the operations on the tympanic membrane, the ossicles and the mastoid process. In the latter part of the book the intracranial complications of suppurative ear disease are dealt with. Of these complications lateral sinus thrombosis is more fully discussed than abscess of the brain and meningitis, the description of the symptoms of the two latter secondary diseases being intentionally brief.

We have been much interested in reading that in the clinic mentioned ossiculectomy has not been found of much value, that Wilde's incision for mastoiditis is no longer made, and that skin-grafting after the radical mastoid operation is hardly thought worth the trouble it involves, and is now rarely undertaken. In the treatment of lateral sinus thrombosis the jugular vein is ligatured only in special circumstances. Operations on the labyrinth for suppuration within it are performed only when the special symptoms of the affection are severe, and when the objective signs, after opening up the tympanum, show that the labyrinth is extensively involved in the suppurative process. For the detection and drainage of cerebral and cerebellar abscesses the mastoid operative route is preferred to that through trephine apertures made in the outer parts of the skull; by the former route the operator follows the course of the disease. Lumbar puncture for diagnostic purposes, a procedure not devoid of danger, is rarely performed, and only when the symptoms present strongly suggest the existence of suppurative meningitis.

We have alluded above to a few of the many interesting problems discussed in the book. Seven good plates illustrate the surgical anatomy of the temporal bone and mastoid operations. The book will be found a valuable one alike by specialists and general surgeons

